# Combined Double Sleeve Lobectomy and Superior Vena Cava Resection for Non-small Cell Lung Cancer with Persistent Left Superior Vena Cava

**DOI:** 10.3779/j.issn.1009-3419.2015.11.10

**Published:** 2015-11-20

**Authors:** Daxing ZHU, Xiaoming QIU, Qinghua ZHOU

**Affiliations:** Department of Lung Cancer Center, West China Hospital, Sichuan University, Chengdu 610041, China

**Keywords:** Lung neoplasms, Sleeve lobectomy, Superior vena cava syndrome, Persistant left superior vena cava

## Abstract

A 65-year-old man with right central type of lung squamous carcinoma was admitted to our department. Bronchoscopy displayed complete obstruction of right upper lobe bronchus and infiltration of the bronchus intermedius with tumor. Chest contrast computed tomography revealed the tumor invaded right pulmonary artery, superior vena cava, and the persistant left superior vena cava flowed into the coronary sinus. The tumor was successfully removed by means of bronchial and pulmonary artery sleeve resection of the right upper and middle lobes combined with resection and reconstruction of superior vena cava (SVC) utilizing ringed polytetrafluoroethylene graft. To the best of our knowledge, this was the first report of complete resection of locally advanced lung cancer involving superior vena cava, right pulmonary artery trunk and main bronchus with persistant left superior vena cava.

## Introduction

Persistent left superior vena cava (PLSVC) is the most common thoracic venous anomaly and usually coexisting with right superior vena cava (SVC) without clinical signs^[1]^. We report an unusual case of non-small cell lung cancer (NSCLC) invading the SVC accompany PLSVC. En bloc resection of the tumor was performed by bronchial and pulmonary artery (PA) sleeve bilobectomy with resection and reconstruction of SVC utilizing ringed polytetrafluoroethylene (PTFE) graft.

## Case report

A 65-year-old man with continuous irritable cough over 15 days was admitted to our department for a mass lesion in the right hilum. He presented with no face swelling and superficial varicose veins. He was a former smoker, and medical history included hypertension and diabetes mellitus. At bronchoscopy, the tumor was found occluding the right upper lobar bronchus and infiltrating the bronchus intermedius. It was diagnosed as squamous cell carcinoma by transbronchial tumor biopsy. Chest contrast computed tomography (CT) revealed that the mass of 80 mm in the maximum diameter located in the right upper lobe. The tumor directly invaded the arch of the right PA and a wide range of SVC. It was discovered that the left brachiocephalic vein (BCV) along the left margin of mediastinum walking down the line directly into the coronary sinus (Fig 1A). A venous flow was detected in the anterolateral descending aorta with dilatation of the coronary sinus (diameter of 21 mm) without other heart abnormalities by transthoracic echocardiography. Based on these findings, the diagnosis of PLSVC was made. There were no detectable metastases in other organs through brain magnetic resonance imaging (MRI), upper abdomen CT scan and bone nuclear scan.

**1:**
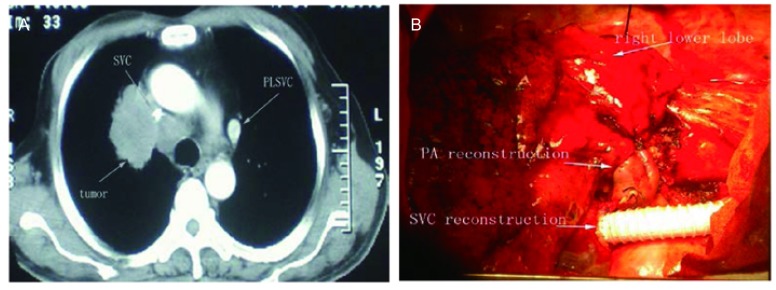
Image of lung cancer and surgery. A: CT scan revealed the tumour infiltration of SVC and the PLSVC; B: The SVC replaced by interpositon of a 12 mm ringed PTFE graft and the sleeve reconstruction of bronchus and PA. CT: computed tomography; SVC: superior vena cava; PLSVC: persistent left SVC.

The right posterolateral thoracotomy was performed on October 31, 2006. As expected, the tumor invaded the wall of SVC and no left brachial cephalic vein bifurcation was observed. The SVC was separated, clamped and resected following intravenous injection of 5, 000 IU of sodium heparin. The ringed PTFE graft of 12 mm in diameter was interpositioned between the right brachial cephalic vein and the origin of SVC by running sutures with 4-0 polypropylene. The tumor resection was completed by double sleeve lobectomy of right upper and middle lobes. The anastomosis between the right main bronchus and the cut end of the lower lobe bronchus was performed by interrupted suture using 3-0 Vicryl (Ethicon, USA). Right PA trunk and right lower PA reconstruction was completed by the running suture with 4-0 polypropylene (Fig 1B). And systematic mediastinal lymph node dissection was performed. All excised margins were tested microscopically negative for malignancy. As anticoagulation treatment, we began with low molecular Heparin on the operative day, then switched and continued to warfarin when thoracic drains removed. The postoperative course was uneventful, and the patient was discharged from the hospital on the 12^th^ postoperative day. Final pathological diagnosis was poorly differentiated squamous cell carcinoma invaded the right SVC, right PA and mediastinal nodes were positive for malignancy.

After 4 cycles of chemotherapy (Gemzar and cisplatin) and 1 cycle of radiotherapy, Chest MRI showed patency of the graft and PLSVC (Fig 2). He died 21 months after operation because of remote metastasis.

**2:**
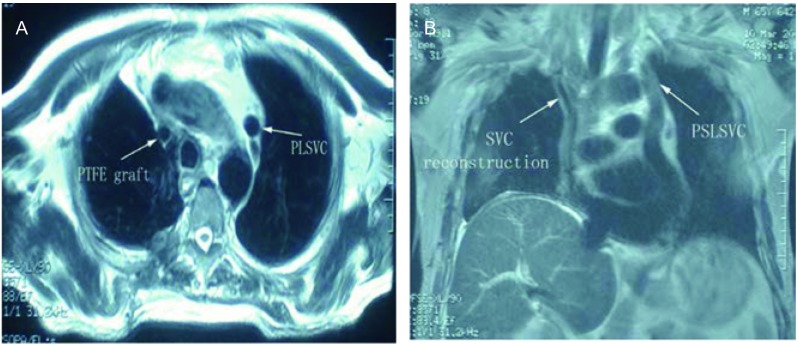
Chest MRI showed patency of the graft of SVC and PLSVC 6 months after surgery. A: chest MRI (cross-sectional); B: chest MRI (sagittal). MRI: magnetic resonance imaging.

## Discussion

PLSVC is an incidental finding in 0.4% of the population and in 3%-8% of patients with congenital heart disease. It usually drains into the right atrium through a dilated coronary sinus, and usually of no clinical significance^[1]^.

The obstruction of blood flow from the head, neck, and arms through the SVC results in the SVC syndrome. Most cases of SVC syndrome are due to malignant tumors, such as lung cancer (80%). The face, neck, and arms were red and swollen, and veins in the chest, neck, and face were dilated^[2]^. But for PLSVC of this case, the patient presented with no face swelling and superficial varicole veins even the SVC nearly completely obstructed by the tumor.

In most instances, locally advanced bronchogenic carcinoma with infiltration of the PA, main bronchus, and SVC was thought to be inoperable. Recently combined extended resections (plasty on the SVC, PA, and main bronchus) in locally advanced NSCLC have been reported occasionally in the literature^[3]^. The outcome was fairly good in some selected patients^[4]^.

For SVC replacement, we routinely set up temporary extracorporeal venous conduit bypass between the right or left internal jugular vein and right or left femoral vein via percutaneous cannulation before operation to prevent from high venous pressure when SVC clamped^[5]^. But in the case of PLSVC, the correct venous bypass should be between the right internal jugular vein and right or left femoral vein. Profiting from the PLSVC, the CVP kept steady and evenly not high to exceed 20 cm H_2_O after SVC clamped. The patient was recovered quickly without major postoperative complications.

In conclusion, we reported a rare locally advance lung cancer invading right PA trunk, the main broncus, and the SVC with PLSVC, successfully treated with extented resections. The PLSVC was considered as collateral vein contributing to drain blood when SVC obstructed by tumor.
